# Methods of crop yield measurement on multi-cropped plots: Examples from Tanzania

**DOI:** 10.1007/s12571-019-00980-5

**Published:** 2019-11-05

**Authors:** Ayala Wineman, C. Leigh Anderson, Travis W. Reynolds, Pierre Biscaye

**Affiliations:** 1 Daniel J. Evans School of Public Policy and Governance, University of Washington, Parrington Hall, 4100 15th Ave NE, Seattle, WA 98195 USA; 2 Department of Community Development and Applied Economics, University of Vermont, Morrill Hall, 146 University Place, Burlington, VT 05405 USA; 3 Department of Agricultural and Resource Economics, University of California at Berkeley, Giannini Hall, Berkeley, CA 94720 USA

**Keywords:** Agricultural systems, Crop yield, Measurement, Tanzania

## Abstract

Precise agricultural statistics are necessary to track productivity and design sound agricultural policies. Yet, in settings where multi-cropping is prevalent, even crop yield—perhaps the most common productivity metric—can be challenging to measure. In a survey of the literature on crop yield in low-income settings, we find that scholars specify how they estimate the area denominator used to measure yield in under 10% of cases. Using household survey data from Tanzania, we consider four alternative methods of allocating land area on multi-cropped plots, ranging from treatment of the entire plot as the yield denominator to increasingly precise approaches that account for the space taken up by other crops. We then explore the implications of this measurement decision for analyses of yield, focusing on one staple crop that is often grown on its own (rice) and one that is frequently found on mixed plots and in intercropped arrangements (maize). A majority (64%) of cultivated plots contain more than one crop, and average yield estimates vary with different methods of calculating area planted—particularly for maize. Importantly, the choice among area methods influences which of these two crops is found to be more calorie-productive per hectare. This choice also influences the statistically significant correlates of crop yield, such that the benefits of intercropping and including legumes on a maize plot are only evident when using an area measure that accounts for mixed cropping arrangements. We conclude that the literature would benefit from greater clarity regarding how yield is measured across studies.

## Introduction

Precise agricultural statistics are necessary to measure and track productivity, allocate scarce resources effectively, and design policies and investments aimed at agricultural sector development in low-income countries. The need for reliable agricultural data has been noted since the 1990s (Kelly et al. 1995; Diskin 1997) and continues to be emphasized to this day (FAO 2010; Carletto et al. 2015a). To address what had been a decline in the quantity and quality of agricultural statistics in low-income countries (and particularly in Africa) (FAO 2010), the Living Standards Measurement Study Integrated Surveys on Agriculture (LSMS-ISA) were launched in 2008 (Carletto et al., 2010). These nationally representative household data sets gather detailed information on agricultural production at the plot level. However, gaps remain in our understanding of how to use household survey data to generate accurate agricultural statistics, with limited attention given to the implications of different choices around how some common variables (such as crop yield) are constructed.

In this paper, we examine the challenge of estimating crop yield on plots that contain more than one crop. Such plots are extremely common in low-income countries, for reasons ranging from risk reduction when crops exhibit different sensitivities to climate variation or disease, to labor constraints, to the maximal utilization of limited space, to the productivity benefits of certain intercropping arrangements (Kelly et al. 1995; FAO 2017).^1^ On mixed plots, area data will be misleading and yields underestimated if the presence of other crops is not accounted for, making it necessary to somehow apportion the cropped area among the component crops. However, no consensus exists on *how* a plot’s land area ought to be attributed to each crop when calculating yields (Fermont and Benson 2011; FAO 2017). Furthermore, agricultural economists often do not specify how they allocate land area among different crops in mixed cropping systems.

Poor or imprecise yield estimates can potentially affect the quality of research on agricultural systems. Thus, a study of the yield effects of climate variability (Rowhani et al., 2011) necessarily rests on having an accurate record of crop yields. Research evaluating the potential for yield improvement (GYGA 2018) similarly requires a clear view of actual yields. Studies of the returns to investments in agricultural research and development often rely on yield measures (Maredia and Raitzer 2010; Perez and Rosegrant 2015), and the calculated benefits of a fertilizer subsidy program are grounded in the measured effects of fertilizer on crop yield (Jayne et al. 2013). Research on the yield effects of intercropping in smallholder systems also hinges on good measures of crop yield (Himmelstein et al. 2017).

There are several options to use as the denominator in a measure of crop yield (quantity harvested per area planted) in the presence of mixed cropping. Analysts may use the whole plot for each crop found on the plot and then report average crop yields by intercropping status, although this does not allow for aggregating crop areas at a higher level (Fermont and Benson 2011). A second option is to divide the area evenly among all crops present on the plot, although the validity of assuming that crops share the land equally is in doubt (Diskin 1997). A third option is to proportionally allocate the plot area among various crops, essentially adjusting the area and yield values to pure stand estimations. Toward this end, the plot area can be divided based on a visual assessment of the proportion occupied by each crop, or with reference to seeding rates or objective measures of row ratios or crop densities (Fermont and Benson 2011; Sud et al. 2016; FAO 2017). The latter methods are considered preferable, though expensive and time-consuming (FAO 2017).

In this paper, we first conduct a survey of the agricultural economics literature to quantify how often authors specify how yield measurements in the presence of multi-cropped plots are constructed. We then consider four alternative methods of allocating land area on mixed plots in Tanzania, focusing on one crop that is often grown on its own (rice) and one that is frequently found on mixed plots and in intercropped arrangements (maize). We ask whether the average crop yield differs with each approach to allocating plot areas; whether the choice of method affects which crop is found to be more productive, or which region of the country is more favorable for a given crop; and whether the statistically significant correlates of crop yield differ in magnitude with different methods. As expected, we find that average yield estimates do vary with different methods of calculating area planted, although this pattern is more pronounced for maize. The choice among methods also influences which of these two staple crops is found to be more calorie-productive per ha, as well as the extent to which inputs, such as fertilizer, are expected to be productivity-enhancing for maize production in Tanzania. Fewer differences are evident when comparing yield estimates with an equal-allocation method versus a more complex proportional scaling method. This suggests that the simpler approach may be adequate when using household survey data.

The remainder of the paper is organized as follows: Section 2 describes the outcome of a survey of the literature to determine how crop areas on multi-cropped plots are typically estimated. Section 3 introduces the data set used in analysis. A description of the variables constructed is provided in section 4, along with an overview of the empirical approach used for econometric analyses. Descriptive and econometric results are found in sections 5 and 6, respectively. Section 7 concludes with a discussion of implications.

## Literature survey

We begin with a survey of the literature to tabulate how authors appear to account for multi-cropping in their yield calculations. We considered the top five most highly-ranked journals in agricultural economics (based on the 2012–2016 average impact factors, per InCites). These include *Food Policy*, *Applied Economic Perspectives and Policy*, the *American Journal of Agricultural Economics*, *Agricultural Economics*, and the *Journal of Agricultural Economics*. Within each journal, a Scopus search was conducted for the term “yield” in the paper title, abstract, and keywords, for all papers published from 2008 to 2017. This produced 222 papers. Among these, we identified those papers that focus on a low- or middle-income country, that refer to household survey data in a quantitative measure of crop yield, and that focus on field crops. This reduced the sample to 40 papers.

Among these, we tabulated how often the paper either uses the entire plot area as the denominator in a yield measure, adjusts the area under a given crop for the presence of other crops on the plot, or does not specify how the yield denominator is defined. We found that three (7.5%) papers specify that they use the entire plot size as a denominator, and none specify explicitly that they adjust the crop areas to account for intercropping. The remaining 37 (92.5%) do not specify what is used as a denominator in the analysis. In some cases, we can discern clues regarding whether the crop area was adjusted to correct for the presence of other crops on the plot. For example, in three papers, intercropped status was included as an explanatory variable in a yield function, with a positive yield effect. This suggests that the yield denominators were likely adjusted. However, in no paper did we find an explanation of *how* crop areas were captured in the survey or adjusted in the analysis.^2^ Thus, it is worthwhile to consider the implications of various choices these authors might have made.

## Data

The National Panel Survey (NPS) of Tanzania is implemented by the Tanzania National Bureau of Statistics and is a research initiative within the Development Economics Research Group of the World Bank. This nationally representative data set captures a rich set of information on farm-household agricultural production at the plot level. We focus primarily on the 2014/15 survey wave, which reflects the main growing season of 2013/14. For this season, the sample contains 2048 households that produced crops. We focus on maize and rice as staple crops that exhibit different cropping patterns. The sample contains 1430 households that produced maize (on 2088 individual plots) and 432 households that produced rice (on 491 plots) in the main season. Some observations are dropped due to incomplete surveys, leaving somewhat smaller sample sizes for analysis. In addition to the estimates of plot area provided by respondents, 71.5% of cultivated plots were measured by Global Positioning Systems (GPS). Population weights are applied in all analyses. The Stata code used in analysis is available from the authors upon request.

## Variables and empirical approach

In the Tanzania NPS, a plot is defined as a contiguous piece of land of uniform tenure (NBS 2017),^3^ and respondents list the crops grown on a given plot and identify whether each crop is intercropped. For seasonal crops, respondents also estimate the proportion of plot area that was cultivated with each crop, with options of one-quarter, one-half, three-quarters, and the entire plot. Where crops are intercropped, however, this estimate necessarily includes the area shared with other crops. Thus, if a 1 hectare (ha) plot is intercropped with maize and beans, both crops are recorded as being planted on 1 ha of land. We focus only on the main growing season in this paper. The proportion of plot area under fruit trees or permanent crops (such as banana or cassava) is not captured in the survey, although respondents do list the number of plants or trees found on the plot.

Several issues arise when estimating the area under each crop. First, the smallest area estimate possible for seasonal crops is one quarter of the plot, which may overestimate the area for marginal crops. Even with no intercropping, the summed area estimates for various crops on a plot can potentially exceed the plot size. Another challenge is that the area under permanent crops can only be estimated with per-plant (or per-tree) area estimates, which are not provided with the data set. Some crops in this category, including fruit trees, are likely to be present on the farm in small numbers, but others may claim a non-negligible amount of space. Finally, it is not obvious how to allocate the land area where multiple crops are intercropped, as the survey does not capture which crops are intercropped together on the same section of the plot, and at what planting density. For example, a plot that is intercropped with one row of maize followed by one row of beans is described in the survey data in an identical manner as another plot that is intercropped with two rows of maize followed by one row of beans.

We examine the implications of four alternative approaches to estimate the area under crops in the presence of mixed cropping (Table 1). Method 1 considers the area under each crop on the plot to be the entire plot area. In cases where more than one crop is present, each crop is assigned the entire plot area. With this crude approach, the summed areas under crops would necessarily exceed the actual plot size whenever more than one crop is present. Note that any fallow land on the plot (which is possible with the plot definition used in the Tanzania NPS) is treated as if it were cropped. Method 2 utilizes the information collected on the proportion of plot area that was planted with a given seasonal crop. As noted, this area may include within it other crops, and as such, the total summed areas under crops may again exceed the plot size. With this method, the area under crops necessarily excludes any fallow areas. Method 3 assumes that the plot area is divided equally among all seasonal crops present, in addition to several permanent crops that are likely to occupy non-negligible areas on multi-cropped plots, including banana and cassava (both staple crops in Tanzania), as well as pigeon pea and pineapple. Other fruit trees / permanent crops are understood to be less likely to affect the area estimates for the crops being evaluated (maize and rice), and this method of allocating areas therefore ignores their presence on the plot. As with method 1, any fallow land on the plot is treated as if it were cropped.

**Table 1:** Methods used to estimate the area under crops

Method	Description
Area 1	The area under crop *i* is considered the area of the entire plot *j*. *Area*1_*ij*_ = *Area*_*j*_
Area 2	The area under seasonal crop *i* is considered the area of plot *j* times the proportion *p*_*ij*_ of plot *j* cultivated with crop *i*, even when crop *i* shares plot *j* with other crops. The area under permanent / tree crop *i* is estimated as the number of plants / trees (*Q*), multiplied by the per-tree (per-plant) areas (*δ*). *For seasonal crops* : *Area*2_*ij*_ = *Area*_*j*_ × *p*_*ij*_ *For permanent*/*tree crops* : *Area*2_*ij*_ = *Q*_*i*_ × *δ*_*i*_
Area 3	The area under crop *i* is considered the area of plot *j* divided by the number of crops planted on the plot. Assumes equal allocation of plot *j* among *n* seasonal or non-seasonal crops (omitting fruit trees, permanent crops, and other crops unlikely to occupy a substantial area). This is not defined for the omitted crops. $Area{3}_{ij}=\frac{Area_j\ }{n_j}$
Area 4	Areas under *m* monocrops *i* are estimated as in Area 2. Areas of *n-m* intercropped crops *i* are estimated and, where these together exceed the residual plot area that is not monocropped, the areas of intercropped crops are scaled down proportionally to the size of the residual (non-monocropped) plot area. *For monocropped crop i* : *Area*4_*ij*_ = *Area*2_*ij*_ $For\ intercropped\ crop\ i: Area{4}_{ij}=\left({Area}_j-{\sum}_{k=1}^{m_j} Area{2}_{kj}\right)\times \left(\frac{Area{2}_{ij}}{\sum_{i=1}^{n_j-{m}_j} Area{2}_{ij}}\right)$ where *k* indexes only monocropped crops on plot *j*

**Fig. 1:**
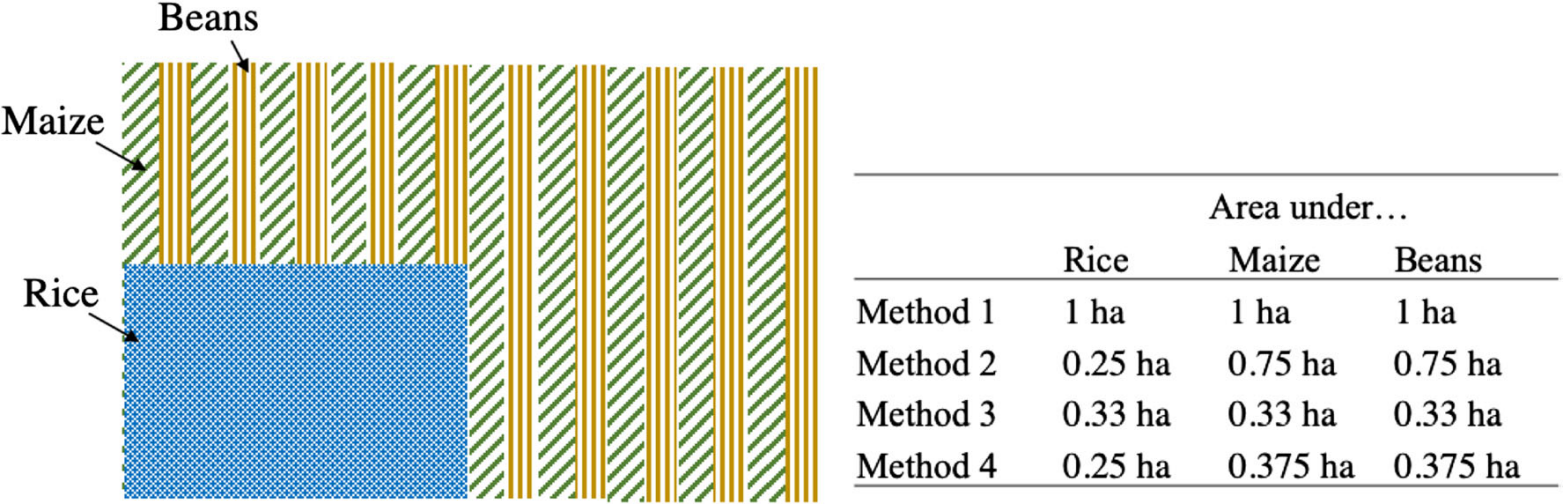
Example of area methods (1 ha plot)

Method 4 accounts for the estimated proportion of the plot on which each seasonal crop is planted and combines this information with per-plant (or per-tree) area estimates for all fruit trees / permanent crops (NBS 2011). Specifically, crops that are monocropped are first assumed to cover the entire quarter-based proportion of plot area (or, in the case of permanent crops, the area estimated using per-plant area estimates). If rice is present on one-quarter of a 1 ha plot and is not intercropped, we assume it covers one-quarter ha (see Fig. 1). Then, the residual area on the plot (not accounted for by monocropped crops) is allocated among the remaining crops. Where the summed area estimates for these crops exceed the residual area on the plot, the area estimates are scaled down proportionally to equal this residual area. In Fig. 1, consider a 1 ha plot with rice that is monocropped on 0.25 ha. In the residual area (not monocropped), maize and beans are intercropped, such that the proportion under each crop is reported as 0.75 ha. These last two values are scaled down to each equal $\frac{0.75}{2}$ ha. Fallow land is accounted for only if the areas under crops sum to less than the plot size.

No method listed above is “perfect,” and even method 4 (utilizing the most detailed information available) is unlikely to be perfectly accurate. However, by applying the four methods to the same data set, we can comment on some of the implications of choosing one method rather than another.

As noted, the size of all plots is estimated by respondents. However, survey respondents often overestimate the area of small plots—particularly by rounding up to an even value, such as one acre—and underestimate the area of large plots (de Groote and Traore 2005; Carletto et al. 2015b). We therefore use the GPS area measures that are available for nearly three-quarters of cultivated plots. The sizes of plots that were not measured are imputed using the respondents’ plot size estimates, along with information derived from local measures of other plots.^4^

The definitions of other variables used in analysis are provided in Table 2. The four approaches to estimating area under crops, as outlined in Table 1, are used to produce four estimates of crop yield (kg harvested / ha under crop). These are numbered 1 through 4, reflecting the methods used to compute area. Input application rates are also computed with reference to crop area estimates 1 through 4. Seed kg/ha uses crop-specific seed amounts and areas (i.e., areas 1–4). However, the use of inorganic fertilizer, manure, and labor were captured only at the plot level, and the denominator for these application rates is the summed area that was cultivated on a given plot. This denominator is the plot size for methods 1 and 3 and also for method 2 whenever the summed areas exceed plot size. For method 4, the per-crop areas are summed within each plot. This approach assumes that fertilizer and labor are allocated equally across crops on a plot, and therefore the application rate applies to every crop observation.

**Table 2:** Variable definitions

Variable	Description
Yield #	Crop yield (kg harvested/ha planted), as estimated with the corresponding area method # (Table 1)
Seed #	Seeding rate (seed kg/ha planted), as estimated with the corresponding area method # (Table 1)
Fertilizer #	Inorganic fertilizer kg per ha cultivated on the plot, as estimated with the corresponding area method # (Table 1)
Manure #	Organic fertilizer kg per ha cultivated on the plot, as estimated with the corresponding area method # (Table 1)
Labor #	Labor days (inclusive of family and hired labor) per ha cultivated on the plot, as estimated with the corresponding area method # (Table 1). Labor includes land preparation/planting and weeding.
1 = Improved seed	Indicator of the use of improved seed
1 = Intercropped	Indicator of whether a crop is intercropped. In this data set, a crop may share a plot with other crops and still be monocropped in its own section.
1 = Legumes	Indicator of whether legumes are present on the plot in the main season
Number crops on plot	Number of crops (inclusive of seasonal crops and fruit trees/permanent crops)
1 = Erosion	Indicator of whether the farmer reports that the plot suffers from erosion
1 = Good soil quality	Indicator of whether the farmer report on the soil quality is “good”, given the options of bad, average, or good
1 = Tractor	Indicator of whether a tractor was used in land preparation
1 = Oxen	Indicator of whether oxen were used in land preparation
1 = Female decision maker(s)	Indicator of whether *only* women are listed as decision-makers for what is grown on a plot

Respondents listed up to two household members that held decision-making power over what to plant on each plot. This information is used to categorize plots by the gender of decision-maker, with categories including women-only or men-only/joint management involving both men and women.

To understand the implications of applying each method to estimate the area under crops, we explore in section 5 a range of descriptive statistics for maize and rice. For example, we compare the mean yield estimates and consider which crop would be viewed as most productive (in terms of calorie production) with each yield measure. In section 6, we also consider whether the detected correlates of crop yield vary with different methods of measuring yield, using a set of linear regressions in which different yield measures are used as the dependent variable.^5^ The following equation is used:

1 $$ {Y}_{ijr}=\alpha +\beta \left[{Area}_{ijr}\right]+{\left[{\boldsymbol{Inputs}}_{\boldsymbol{ijr}}\right]}^{\prime}\boldsymbol{\delta} +{\left[\boldsymbol{Plot}\_{\boldsymbol{characteristics}}_{\boldsymbol{jr}}\right]}^{\prime}\boldsymbol{\theta} +{\tau}_r+{\varepsilon}_{ijr} $$

where *Y*_*ijr*_ is a measure of crop yield for crop *i* on plot *j* in region *r*; *Area*_*ijr*_ is the estimate of area under the crop that corresponds to the method used to estimate *Y*_*ijr*_; ***Inputs***_***ijr***_ is a vector of corresponding input intensities; ***Plot*** _ ***characteristics***_***jr***_ is a vector of plot characteristics; *τ*_*r*_ are region fixed effects; and *ε*_*ijr*_ is a stochastic term. The region fixed effects control for broad geographic differences in seasonal weather outcomes in this growing season. This model is estimated separately for maize and rice.^6^

## Descriptive results

In the 2013/14 main season in Tanzania, 64% of cultivated plots contain more than one crop, and because plots containing a single crop tend to be smaller than others, roughly three-quarters of the area under crops (depending on which area method is used) contains multiple crops. This makes the choice of how to allocate plot area among multiple crops highly consequential when generating agricultural statistics. Descriptive statistics in Table 3 illustrate some of these consequences for maize and rice. As expected, the estimated area under each crop is adjusted downward over areas 2–4 to reflect, in different ways, the presence of other crops on the plot and the plot areas that are left uncultivated. It follows that estimated yields will generally increase when using these methods.

**Table 3:** Summary statistics

		Maize		Rice	
	Variable	Mean	SD	Mean	SD
Area planted (ha)	Area 1	1.44	2.97	1.32	2.85
	Area 2	0.94	1.85	0.87	1.43
	Area 3	0.67	1.25	0.87	1.29
	Area 4	0.66	1.20	0.82	1.26
Crop and plot characteristics	1 = Plot contains only this crop	0.21	0.41	0.73	0.44
	Number crops on plot	3.18	2.30	1.75	1.73
	1 = Plot contains permanent crops	0.45	0.50	0.15	0.36
	1 = Crop is intercropped	0.66	0.47	0.15	0.35
	1 = Legumes found on plot	0.40	0.49	0.05	0.22
	1 = Improved seed	0.46	0.50	0.09	0.29
	1 = Plot suffers from erosion	0.16	0.37	1.90	0.30
	1 = Soil quality is good	0.44	0.50	0.52	0.50
	1 = Tractor used in land preparation	0.07	0.25	0.15	0.35
	1 = Oxen used in land preparation	0.38	0.48	0.47	0.50
	1 = Female decision makers only	0.25	0.43	0.26	0.44
**Input intensities**					
Seed kg / ha	Seed 1	17.61	31.96	53.74	58.75
	Seed 2	21.67	38.20	61.34	65.95
	Seed 3	36.55	70.44	64.30	74.76
	Seed 4	36.80	140.60	64.08	67.77
Fertilizer kg / ha	Fertilizer 1 and 3	20.06	56.27	12.06	50.67
	Fertilizer 2	20.25	56.63	12.18	50.95
	Fertilizer 4	20.47	56.98	12.21	51.06
Manure kg / ha	Manure 1 and 3	249.13	888.58	47.37	306.79
	Manure 2	250.45	893.11	48.27	311.62
	Manure 4	253.88	901.60	48.44	311.86
Labor days / ha	Labor 1 and 3	130.10	217.32	178.62	236.01
	Labor 2	132.40	217.81	189.04	242.96
	Labor 4	135.18	218.04	190.30	242.94
	Observations	2028		469	

Several other plot characteristics are relevant to yield. It is much more common for rice to be planted on its own, with 73% of rice observations on pure stand plots, while just 21% of maize observations are pure stand. We therefore expect the different decisions regarding area estimates under each crop to be more relevant for maize than for rice. Because legumes fix nitrogen, they are understood to enhance the yield of other crops when intercropped together (Dakora and Keya 1997; Snapp et al. 2010). In 40% of maize cases, a legume is also found on the plot. (The definition of a “plot” in this survey, which can include multiple cropping regimes, leaves us uncertain as to whether an intercropped maize crop is intercropped directly with the legume or is planted adjacent to it.)

As shown in Table 4, mean yield estimates generally increase over yields 1–4. Particularly for maize, accounting for the space taken by other crops on the plot (yields 3 and 4) produces much higher average yields than are otherwise estimated. In the bottom panel of Table 4, we test whether the mean yields that are derived with different methods are statistically significantly different. For maize, it seems that all four methods produce mean values that differ from one another. For rice, yield 1 (using the entire plot size as the area under crop) does differ significantly from the other methods. However, the other three yield measures do not significantly differ from one another, indicating that a decision among these may not be very consequential for analyses of rice yield.

**Table 4:** Comparison of mean yields (kg/ha) with different area measures

	Yield 1		Yield 2		Yield 3		Yield 4	
	Mean	SD	Mean	SD	Mean	SD	Mean	SD
Maize	1066.95	1471.34	1292.45	1664.48	1992.01	2354.87	1865.02	2228.11
Rice	1553.82	1667.09	1755.63	1701.81	1803.24	1776.26	1810.00	1709.99
		Tests (*P*-values)^a^						
		1 = 2	1 = 3	1 = 4	2 = 3	2 = 4	3 = 4	
	Maize	**0.000**	**0.000**	**0.000**	**0.000**	**0.000**	**0.078**	
	Rice	**0.067**	**0.027**	**0.020**	0.675	0.626	0.953	

^a^P-values from two-sample t-tests. Values of <0.1 are in bold font

Policy makers, agricultural research stations, or development practitioners may decide to prioritize one crop over the other based on an understanding of their relative productivity. We next consider whether relative productivities differ with different approaches to measuring yield. Because kilograms cannot readily be compared across crops, productivity is measured here as the mean calories produced per ha cultivated, with per-kg calorie values taken from Wu Leung et al. (1968). The results in Table 5 show that, while rice is estimated to be more productive with yields 1 and 2, maize emerges as the more productive crop with yields 3 and 4. Because maize is so often grown with other crops, its superior productivity is obscured until the space claimed by other crops on the plot is somehow addressed.

**Table 5:** Calorie productivity across crops

	Calories per ha (mean yield * calories/kg)		
	Maize 3570 cal/kg	Rice 3440 cal/kg	Most calorie-productive crop
Yield 1	3,809,012	5,547,130	Rice
Yield 2	4,614,047	6,267,585	Rice
Yield 3	7,111,497	6,437,567	Maize
Yield 4	6,658,121	6,461,696	Maize

Policy makers may also allocate resources to different geographic regions with consideration of their relative productivity for a given crop. We next ask, does this calculus shift with different yield measures? Table 6 displays the mean maize yields found in two of the primary maize-producing zones in Tanzania, the Southern Highlands and Northern zone. In Northern zone, maize is somewhat more likely to be intercropped (60%) and to have a higher number of crops sharing the plot (mean = 3.34). For the Southern Highlands, these figures are 55% and 2.64, respectively. For all four yield measures, the Southern Highlands exhibit higher average maize yields. However, for yield 1, this value is 20.86% higher than the average value observed in the Northern zone, while it is just 4.90% higher with yield 3. Furthermore, the differences in mean yields are not statistically significant when using yield 3 or yield 4. Therefore, the extent to which the Southern Highlands are found to be more favorable for maize production does vary, depending on how yield is measured.

**Table 6:** Comparison of maize yield across zones

	Yield (kg/ha)						
	Northern zone (N)		Southern Highlands (SH)		Test (N = SH)^a^		
	Mean	SD	Mean	SD	Difference in mean (SH - N)	P-value	% difference (SH vs. N)
Yield 1	1417.98	1500.41	1713.73	1929.69	295.75	0.036	20.86%
Yield 2	1741.74	1672.18	2021.35	2137.66	279.61	0.074	16.05%
Yield 3	2682.13	2582.45	2813.69	2747.40	131.56	0.538	4.90%
Yield 4	2460.66	2404.89	2712.37	2626.02	251.73	0.213	10.23%
Observations	268		392				

^a^T-test for equality of mean values in Northern zone and Southern Highlands

**Fig. 2:**
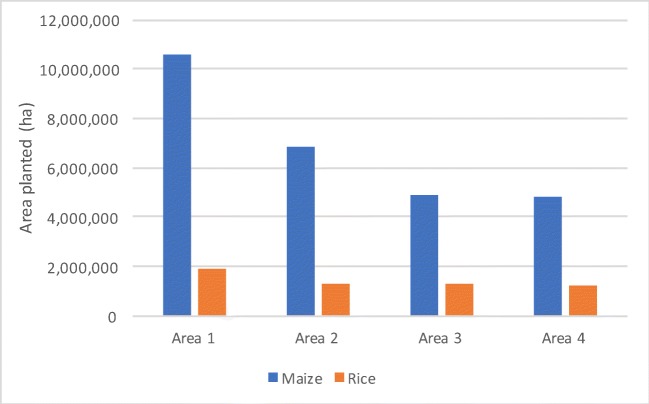
Area under crops (2013/14 main season)

Because some area measures do not account for mixed cropping, different measures will necessarily produce divergent stories regarding the aggregate areas found under each crop. (In fact, the commonly-cited FAOSTAT database (FAO 2019) does not seem to provide guidance for how countries should report the area harvested of a given crop, defining this only as “the area from which a crop is gathered.”) Using the Tanzania NPS data, Fig. 2 displays the total areas cultivated with maize and rice in the main season. Methods 1 and 2 necessarily double-count areas that are shared by multiple crops, resulting in high country-level area estimates for each crop. Thus, method 2 results in an aggregate area under maize that is 42.38% greater than method 4. For rice, which is usually planted alone, this difference is just 6.48%. Because method 3 assumes the entire plot is cultivated, the estimated total area cropped is a bit larger than for method 4.

We next consider possible variation in farm and management factors correlated with yield using two measures that vary dramatically from one another, area 2 (the un-adjusted share of plot area under a given crop) and area 4 (with crop areas that have been scaled down proportionally to plot size). Table 7 presents the average yields for these two measures for various subgroups of crop observations. The average difference between yield 4 and yield 2 for a given subgroup is given in the last column, with asterisks that denote a statistically significant difference in this value between the two mutually exclusive subgroups. For example, for intercropped maize observations, the average difference between yield estimates is 798.23 kg/ha, and this is statistically significantly different (at the 1% level) from the average difference seen for monocropped observations (134.85 kg/ha).

**Table 7:** Yield estimates (kg/ha) among plot subgroups (using area estimates 2 and 4)

		Yield 2		Yield 4		Difference in means^a^
	Observations	Mean	SD	Mean	SD	Yield 4 – Yield 2
**Maize**						
Plot contains only this crop	389	1632.94	1892.10	1632.94	1892.10	0.00***
Plot contains other crops	1639	1203.92	1588.78	1925.36	2304.20	721.44
Crop is monocropped	644	1535.74	1748.50	1670.59	1952.55	134.85***
Crop is intercropped	1384	1167.02	1605.81	1965.26	2352.06	798.23
Plot is small (≤ 0.5 ha)	1649	1431.11	1762.35	2043.54	2322.14	612.43***
Plot is large (> 0.5 ha)	379	681.09	914.48	1077.92	1524.88	396.83
Men are involved in decision-making on the plot	1526	1319.80	1714.04	1876.11	2256.36	556.32
Only women decide what to plant on the plot	502	1210.99	1505.85	1831.97	2143.59	620.98
**Rice**						
Plot contains only this crop	333	1965.27	1777.12	1965.27	1777.12	0.00***
Plot contains other crops	136	1179.93	1319.19	1383.62	1432.17	203.68
Crop is monocropped	402	1874.95	1770.70	1889.63	1769.31	14.68***
Crop is intercropped	67	1054.27	965.19	1341.98	1216.41	287.71
Plot is small (≤ 0.5 ha)	423	2002.21	1746.48	2052.52	1748.15	50.30
Plot is large (> 0.5 ha)	46	789.37	1065.01	859.69	1135.12	70.32
Men are involved in decision-making on the plot	341	1785.22	1660.08	1832.33	1653.71	47.12
Only women decide what to plant on the plot	128	1672.01	1818.82	1746.89	1865.37	74.88

^a^Asterisks denote the statistical significance level of a Wald test for equality of mean values. *** *p* < 0.01, ** *p* < 0.05, * *p* < 0.1

As expected, the difference between yield 4 and yield 2 is larger, on average, for crop observations that are found on plots that contain other crops, relative to those with only one crop. For maize, the average difference between yield measures is larger on small plots (≤ 0.5 ha) than on large plots. Descriptively, this suggests that the inverse area-productivity relationship that is commonly observed in developing countries (Larson et al. 2014; Wineman and Jayne 2017) would be more intense when measured with yield 4, as compared to yield 2. For both maize and beans, the average difference in yield estimates between yield 4 and yield 2 is slightly smaller for plots on which men are involved in decision-making, as compared with those on which only women are involved. However, this difference in the average difference is never statistically significant. This suggests that any detected gender gap in crop production would likely be unaffected by the alternative methods used to measure yield.

## Econometric results

We now apply an econometric analysis to understand whether the method applied for crop area estimation also influences our understanding of the most important correlates of crop yield. Equation (1) is used for each area method, in turn. Recall that the different area methods affect the dependent variable (yield), as well as the estimates for area planted and seed, fertilizer, manure, and labor intensities.

Results for maize yield are given in Table 8. An informal comparison of coefficients across columns suggests that the intensity of the inverse area-productivity relationship is greater (the magnitude of the coefficient is larger) for methods 3 and 4, as compared with methods 1 and 2. Thus, another ha planted is associated with a decrease in maize yield of 28.31 kg/ha (when using method 1) or 164.27 kg/ha (when using method 4). This is consistent with the descriptive results of Table 4. The relationship between seeding rate and yield also varies, depending on the method used. Thus, method 3 indicates that another kg of maize seed/ha is associated with a yield increase of 9.59 kg/ha, though this coefficient is much smaller (2.13) and not statistically significant in column 4. This could have implications for the optimal seeding rate suggested by agricultural extension agents. The coefficient on the use of improved seed varies considerably across equations (by up to 55%), and this may have implications for the estimated returns to maize seed intensification in this setting (Arslan et al. 2015; Komarek et al. 2018). Likewise, the relationship between fertilizer application rate and maize yield also varies with different area methods, a point which is likely to affect analyses of fertilizer profitability (e.g., Burke et al. 2017; Komarek et al. 2018; Liverpool-Tasie 2017; Theriault et al. 2018).

**Table 8:** Correlates of maize yield (OLS)

	(1)	(2)	(3)	(4)
	Dependent variable: Yield (kg/ha)			
	Yield 1	Yield 2	Yield 3	Yield 4
Area 1 (ha)	−28.31***			
	(0.00)			
Kg seed / ha (area 1)	14.41***			
	(0.00)			
Kg fertilizer / ha (area 1)	5.86***			
	(0.00)			
Kg manure / ha (area 1)	0.06			
	(0.19)			
Labor days / ha (area 1)	1.86***			
	(0.00)			
Area 2 (ha)		−49.69**		
		(0.01)		
Kg seed / ha (area 2)		13.24***		
		(0.00)		
Kg fertilizer / ha (area 2)		6.28***		
		(0.00)		
Kg manure / ha (area 2)		0.08		
		(0.13)		
Labor days / ha (area 2)		1.72***		
		(0.00)		
Area 3 (ha)			−165.85***	
			(0.00)	
Kg seed / ha (area 3)			9.59***	
			(0.00)	
Kg fertilizer / ha (area 3)			9.25***	
			(0.00)	
Kg manure / ha (area 3)			0.11	
			(0.15)	
Labor days / ha (area 3)			2.34***	
			(0.00)	
Area 4 (ha)				−164.27***
				(0.00)
Kg seed / ha (area 4)				2.13
				(0.15)
Kg fertilizer / ha (area 4)				8.48***
				(0.00)
Kg manure / ha (area 4)				0.14**
				(0.03)
Labor days / ha (area 4)				3.41***
				(0.00)
1 = Improved seed used	281.32***	361.59***	436.86***	413.01***
	(0.00)	(0.00)	(0.00)	(0.00)
1 = Crop was intercropped	−238.85***	−189.09***	264.23**	209.58**
	(0.00)	(0.01)	(0.03)	(0.04)
1 = Legumes found on the plot	37.79	153.26	609.17***	612.69***
	(0.65)	(0.10)	(0.00)	(0.00)
1 = Problems with erosion	−211.44***	−236.97**	−299.75**	−266.48*
	(0.01)	(0.02)	(0.03)	(0.09)
1 = Soil quality is good	247.82***	268.08***	376.09***	365.44***
	(0.00)	(0.00)	(0.00)	(0.00)
1 = Tractor used in in land preparation	550.13***	582.66***	1005.10***	994.65***
	(0.00)	(0.00)	(0.00)	(0.00)
1 = Oxen used in in land preparation	237.79*	228.65*	286.32*	287.52*
	(0.06)	(0.09)	(0.09)	(0.10)
1 = Only women decide what to plant on this plot	−144.42**	−211.72***	−282.47***	−277.99***
	(0.02)	(0.00)	(0.00)	(0.00)
Region fixed effects	Y	Y	Y	Y
Constant	−103.19	−93.44	−233.95	−174.67
	(0.34)	(0.50)	(0.28)	(0.38)
Observations	2028	2028	2028	2028
R-squared	0.481	0.429	0.417	0.391

P-values in parentheses; *** p < 0.01, ** p < 0.05, * p < 0.1

Using methods 1 and 2, which do not adjust for intercropping, it would appear that intercropping is negatively correlated with maize yield. However, once we account for the presence of multiple crops on the same plot, intercropping appears to be beneficial for yields. Along the same lines, the positive yield effect of having legumes present on the plot is not evident (in a statistically significant manner) until columns 3–4, when it becomes clear that planting maize alongside legumes is associated with higher yields on the order of 600 kg/ha. This is consistent with research on the maize yield benefits of intercropping with legumes (Snapp et al. 2010; Arslan et al. 2015; Droppelmann et al. 2017). The coefficient on having only women as plot managers is negative and statistically significant across all models, and this is consistent with other research that finds a gender productivity gap in agriculture (Peterman et al. 2011; Slavchevska 2015).^7^

The same exercise is repeated for rice yield in Table 9. Again, the inverse relationship between area and yield emerges as least intense when using method 1. Again, intercropping initially appears to be detrimental to rice yield (columns 1–2) in a statistically significant manner until we account for the space taken up by other crops. Surprisingly, the use of improved rice seed is not statistically significantly associated with yield, although Wineman et al. (2019) suggest that farmers’ identification of improved varieties may not always be correct.

**Table 9:** Correlates of rice yield (OLS)

	(1)	(2)	(3)	(4)
	Dependent variable: Yield (kg/ha)			
	Yield 1	Yield 2	Yield 3	Yield 4
Area 1 (ha)	−51.94**			
	(0.02)			
Kg seed / ha (area 1)	7.10***			
	(0.00)			
Kg fertilizer / ha (area 1)	6.15***			
	(0.01)			
Kg manure / ha (area 1)	0.06			
	(0.72)			
Labor days / ha (area 1)	0.83			
	(0.19)			
Area 2 (ha)		−150.97***		
		(0.01)		
Kg seed / ha (area 2)		4.89***		
		(0.01)		
Kg fertilizer / ha (area 2)		5.99***		
		(0.01)		
Kg manure / ha (area 2)		0.19		
		(0.36)		
Labor days / ha (area 2)		0.81		
		(0.19)		
Area 3 (ha)			−201.39***	
			(0.00)	
Kg seed / ha (area 3)			5.00**	
			(0.04)	
Kg fertilizer / ha (area 3)			5.98***	
			(0.01)	
Kg manure / ha (area 3)			0.29	
			(0.26)	
Labor days / ha (area 3)			0.82	
			(0.21)	
Area 4 (ha)				−177.54***
				(0.00)
Kg seed / ha (area 4)				5.13***
				(0.01)
Kg fertilizer / ha (area 4)				5.82***
				(0.01)
Kg manure / ha (area 4)				0.18
				(0.35)
Labor days / ha (area 4)				0.77
				(0.21)
1 = Improved seed used	−267.30	−224.24	−150.45	−162.67
	(0.40)	(0.47)	(0.64)	(0.61)
1 = Crop was intercropped	−428.27***	−381.35**	−56.29	−220.78
	(0.00)	(0.01)	(0.76)	(0.17)
1 = Legumes found on the plot	−276.41	192.15	676.22*	255.14
	(0.14)	(0.58)	(0.05)	(0.41)
1 = Problems with erosion	−118.43	21.18	−144.50	−20.34
	(0.70)	(0.95)	(0.66)	(0.95)
1 = Soil quality is good	197.68	217.89	248.49	249.13
	(0.15)	(0.14)	(0.11)	(0.11)
1 = Tractor used in in land preparation	105.56	71.32	−20.63	65.74
	(0.72)	(0.81)	(0.95)	(0.83)
1 = Oxen used in in land preparation	118.75	100.27	210.97	114.80
	(0.65)	(0.70)	(0.45)	(0.66)
1 = Only women decide what to plant on this plot	−385.03**	−374.64*	−331.02*	−358.08*
	(0.03)	(0.05)	(0.09)	(0.07)
Region fixed effects	Y	Y	Y	Y
Constant	1579.29***	1678.90***	1630.85***	1649.62***
	(0.00)	(0.00)	(0.00)	(0.00)
Observations	469	469	469	469
R-squared	0.451	0.385	0.367	0.373

P-values in parentheses; *** p < 0.01, ** p < 0.05, * p < 0.1

We next test for statistically significant differences in coefficients across different columns of Tables 8, 9. In a seemingly unrelated estimation of yield functions, we compare the coefficients from column 2 and column 4 (Table 10, top panel). The P-values reported indicate that, across these two models, the coefficients often differ for maize yield, though they rarely differ for rice. It seems clear that, for crops that are often grown on mixed plots, the method applied to estimate area under the crop will affect an analyst’s conclusions regarding the correlates of crop yield and especially the magnitude of these relationships. In the lower panel of Table 10, the same exercise is repeated with the coefficients from columns 3 and 4. In this exercise, we see far fewer small P-values, indicating fewer instances where the magnitude of the coefficients differs across models in a statistically significant manner. This suggests that even the relatively simplistic approach of method 3 may be adequate to discern the correlates of yield.

**Table 10:** Tests for difference in coefficients across models with different area measures

	Test: *β*(Eq. 2) = *β*(Eq. 4) *P*-values	
	Maize	Rice
Area (ha)	**0.000**	0.163
Kg seed / ha	**0.001**	0.570
Kg fertilizer / ha	**0.004**	0.265
Kg manure / ha	0.192	0.723
Labor days / ha	**0.000**	0.472
1 = Improved seed used	0.405	0.372
1 = Crop was intercropped	**0.000**	**0.019**
1 = Legumes found on the plot	**0.000**	0.622
1 = Problems with erosion	0.734	**0.055**
1 = Soil quality is good	**0.062**	0.149
1 = Tractor used in in land preparation	**0.037**	0.577
1 = Oxen used in in land preparation	0.489	0.917
1 = Only women decide what to plant	0.201	0.675
	Test: *β*(Eq. 3) = *β*(Eq. 4) P-values	
	Maize	Rice
Area (ha)	0.918	0.492
Kg seed / ha	**0.000**	0.932
Kg fertilizer / ha	0.221	0.957
Kg manure / ha	0.526	0.264
Labor days / ha	**0.000**	0.879
1 = Improved seed used	0.609	0.876
1 = Crop was intercropped	0.260	0.123
1 = Legumes found on the plot	0.956	**0.001**
1 = Problems with erosion	0.607	0.308
1 = Soil quality is good	0.798	0.976
1 = Tractor used in in land preparation	0.918	0.449
1 = Oxen used in in land preparation	0.982	0.750
1 = Only women decide what to plant	0.922	0.515

Note: P-values of less than 0.1 are denoted in bold font, indicating a statistically significant difference between coefficients in the two models being compared

## Conclusion

This paper is motivated by the question of whether construction choices for a very common metric of productivity—crop yield—could affect empirical analyses and thereby have policy and investment implications (Anderson et al. 2015). We examine several questions of relevance to policy makers and development partners. For example, when allocating scarce resources earmarked for agricultural development, which crop is more productive? Which region of the country is associated with the highest returns for a given crop? And what is the relationship between certain inputs and crop yield, with implications for economically sound strategies to promote crop production?

We generally find that the method used to assign area (the denominator in crop yield) to a given crop does affect the conclusions derived regarding yield patterns and correlates of higher yields. The most obvious difference is simply around the estimation of average yields, which increase once the space taken by other crops is deducted from the denominator. This is less relevant for rice, which is often grown on its own, than for maize. As a result, cross-crop comparisons of productivity will produce different “winners”, depending on how yield is measured. Another obvious consequence of the choice among area methods is the very different views that emerge regarding the aggregate emphasis on different crops, in terms of production areas. Specifically, the gap between areas devoted to maize versus rice production in Tanzania is much reduced when accounting for the mixed cropping arrangements that are most common for maize.

We further find that, without accounting for the space taken up by other crops on the plot, the anticipated benefits of intercropping and especially including legumes on a maize plot may not be evident. And while the statistical significance of various correlates of crop yield are often consistent, regardless of how crop area is captured, the magnitude of these relationships does shift. This may affect the extent to which a particular input, such as fertilizer used in maize production, appears to be profitable for crop-producing households. Interestingly, the results are fairly (though not perfectly) consistent when a crop’s area is estimated with method 3 and method 4. When detailed information is not available, it seems that the simpler approach—equal division of the plot area among crops present— may be acceptable for this type of basic analysis. Furthermore, the information used in method 3 likely represents a lighter reporting burden for respondents.

It should be noted that the method used to allocate plot area among crops is not the only challenge related to estimating crop yields from household surveys. The approach to plot area measurement can also shift yield estimates (Carletto et al. 2015b). In addition, because some planted area may be left unharvested for reasons that extend beyond production failures, such as theft, wildfire, or wildlife pests (Anderson et al. 2015), analysts may want to use area harvested rather than area planted in the denominator. Furthermore, there is some debate regarding how the numerator in crop yield (total quantity harvested) should be estimated. While attention has been given to the possibility that farmer estimates may be biased (Gourlay et al. 2017), other authors note that crop cuts may also be biased or produce results that are not representative of the entire plot (especially when planting densities are uneven), or may be inconsistent with what a farmer would consider to be harvestable (Diskin 1997; Fermont and Benson 2011; Sud et al. 2016). It can be especially difficult to estimate harvests from crops with an extended harvest period, such as cassava, or to identify the area claimed by crops that are produced more than once in a growing season. It is unclear how crop byproducts, such as the harvesting of pumpkin leaves in addition to the fruit produced, should be treated in yield estimates (Kelly et al. 1995). Finally, it is unclear how to aggregate portions of harvest that are of different forms because the harvests took place at different times during the season. For example, maize in Tanzania is often partly collected before the main harvest and consumed as “green maize”.

A final lesson of this paper is that readers should be somewhat cautious when comparing results of yield research, as different authors may make different construction decisions for estimating yield. This applies to all topics mentioned in the previous paragraph, in addition to decisions around how to account for the presence of multiple crops on a plot. Given our finding that construction decisions can influence the results of analysis, it follows that the literature would benefit from greater clarity regarding *how* yield is measured across studies.

## Footnotes

1 The term “mixed cropping” refers to multiple crops being grown together, often with their seed mixed before being broadcast. In contrast, “intercropping” refers to multiple crops being cultivated in a definite pattern (Kelly et al. 1995; FAO 2017). In this paper, we use the terms “multi-cropping” or “mixed plots” to refer to multiple crops being found on the same plot, regardless of their arrangement.

2 Note that mixed cropping / intercropping may not be relevant for all crops. In particular, irrigated rice production may not be conducive to intercropping. When all studies of rice yield are removed from the analysis, we are left with 29 papers, of which 27 (93.10%) do not specify how they estimate the area under the crop being studied.

3 In some other surveys, the definition of an agricultural “plot” refers to an area of uniform crop management, such as monocrop or an intercrop regime that is uniform throughout the plot area. Unlike in the Tanzania NPS, respondents in the Uganda LSMS-ISA report a continuous percent of plot area allocated to each crop, and permanent crops are treated as any other crop when estimating areas. Thus, the challenges associated with measuring crop yield are somewhat unique to each survey.

4 Specifically, we divide plot size estimates into “units”, such as one-quarter acre, one-half acre, or five acres. Thus, a plot that is listed as being 0.75 acres is understood to be comprised of one and a half one-half acre units. The median size of each unit is captured using the GPS measures of other plots, and we apply the median value from the smallest local geographic unit for which 10 measurements have been taken. If a plot is estimated as 0.75 acres, and the median measured value of a one-half acre unit in the same enumeration area is 0.3 acres, then the plot size is imputed as 0.45 acres.

5 Although nonlinear production functions are often used, a simple, linear model is preferred here for clarity and for purposes of illustration (consistent with Liverpool-Tasie (2017)).

6 Note that crop yield observations are specific to seed type, such that if two seed types for the same crop are grown on the same plot, it is treated as two observations.

7 A robustness check using a Cobb-Douglas production function leads to a similar conclusion that the approach to estimating area can sometimes influence the input elasticities in a statistically significant manner.
